# 722. Risk Factors for Infection or Colonization with Ceftolozane/Tazobactam Non-Susceptible Extensively β-Lactam Resistant *Pseudomonas aeruginosa*

**DOI:** 10.1093/ofid/ofad500.783

**Published:** 2023-11-27

**Authors:** Michelle Lee, Jerod Nagel, Walaiporn Wangchinda, Virginia M Pierce, Aaron Smith, Olivia Bishop, Jason M Pogue

**Affiliations:** Rhode Island Hospital, Providence, Rhode Island; Michigan Medicine, Ann Arbor, Michigan; University of Michigan College of Pharmacy, Ann Arbor, Michigan; University of Michigan Medical School; Michigan Medicine, Ann Arbor, Michigan; Michigan Medicine, Ann Arbor, Michigan; University of Michigan College of Pharmacy, Ann Arbor, Michigan; University of Michigan, College of Pharmacy, Ann Arbor, Michigan

## Abstract

**Background:**

Treatment of infections due to multidrug resistant (MDR) *Pseudomonas aeruginosa* is complicated by co-resistance amongst traditional anti-pseudomonal β-lactams, as 50% of MDR isolates exhibit extensive β-lactam resistance (EBR, non-susceptible to cefepime, ceftazidime, piperacillin/tazobactam, and meropenem). Ceftolozane/tazobactam (C/T) was developed to combat MDR and EBR *P. aeruginosa*. Unfortunately, C/T resistance has proliferated. We designed this retrospective case-control study to identify risk factors for C/T non-susceptibility (NS) amongst EBR *P. aeruginosa*.

**Methods:**

Between 2015 to 2020, hospitalized adult patients infected or colonized with EBR *P. aeruginosa* were identified. C/T susceptibility was defined per the 2023 Clinical and Laboratory Standards Institute (CLSI) guidelines (susceptible (S) if minimum inhibitory concentration (MIC) ≤ 4 mg/L, NS if MIC ≥ 8 mg/L). Patients with an EBR *P. aeruginosa* NS to C/T were classified as cases, whereas those with S isolates were controls. Patients with multiple EBR *P. aeruginosa* isolates over the study period were only included once. If a patient had a C/T NS isolate, they were classified as cases and the first C/T NS isolate was included as the index culture. If the patient did not have a C/T NS isolate over the study period, they were considered a control and the first C/T S isolate was the index culture. Bivariate and multivariate modeling was performed to identify independent predictors for C/T NS.

**Results:**

188 unique patients with C/T susceptibility performed were included in the study; 96 (51%) were C/T NS and 92 (49%) were C/T S. Table 1 shows the bivariate comparisons of key baseline characteristics between groups. Independent predictors of C/T NS are displayed in table 2.

**Figure:**
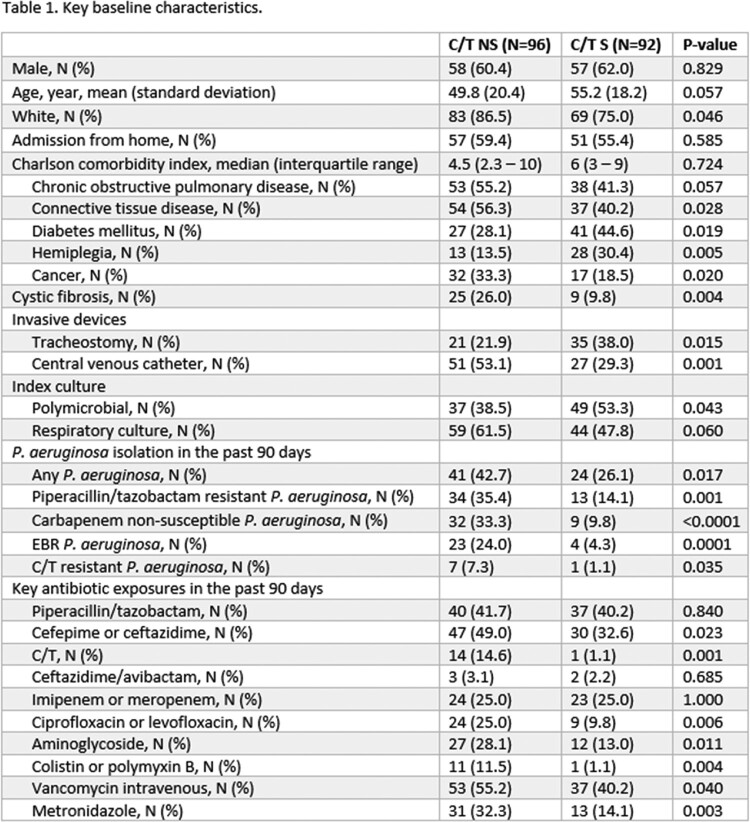

**Figure:**
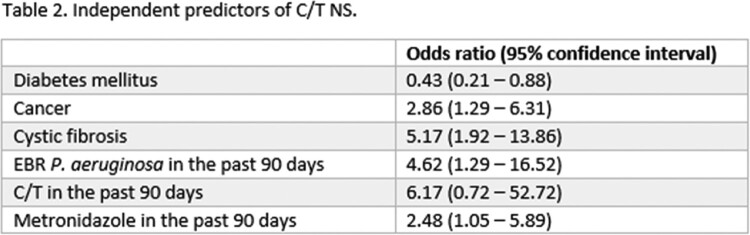

**Conclusion:**

Independent predictors for C/T NS amongst EBR *P. aeruginosa* isolates were a history of cancer, cystic fibrosis, and isolation of EBR *P. aeruginosa* in the past 90 days. While receipt of cefepime or ceftazidime in the past 90 days was associated with C/T NS in bivariate comparisons, the association did not remain in multivariate modeling. C/T exposure was not a statistically significant risk factor, but it was numerically associated with NS and the lack of significance was likely due to the small number of patients (n = 15) who received it.

**Disclosures:**

**Virginia M. Pierce, MD, FIDSA**, UpToDate, Inc.: Authorship royalties **jason M. Pogue, PharmD**, AbbVie: Advisor/Consultant|Entasis: Advisor/Consultant|Ferring: Advisor/Consultant|GSK: Advisor/Consultant|Merck: Advisor/Consultant|Merck: Grant/Research Support|Qpex: Advisor/Consultant|Shionogi: Advisor/Consultant

